# Investment and financing maturity mismatch: Moderating role of financialization in regulatory context for A-share listed Chinese companies

**DOI:** 10.1016/j.heliyon.2024.e34488

**Published:** 2024-07-11

**Authors:** Hongwen Jia, Nan Gao, Fayyaz Ahmad, Ahsan Farooq, Aamir Javed

**Affiliations:** a School of Economics, Lanzhou University, Lanzhou, 730000, Gansu, China; b School of Economics, University of Sahiwal, Sahiwal, 57000, Punjab, Pakistan; c Department of Economia Aziendale, University G'd Annunzio of Chieti and Pescara, Italy

**Keywords:** Corporate financialization, Investment and financing maturity mismatch, Financial regulation, Moderating effect

## Abstract

As the financialization issue is getting more and more attention, the behavioral motives and effects behind this appearance should not be ignored, and it is of great practical significance for the high-quality development of China's real economy to explore the impact and mechanism of the financialization trend on the investment and financing maturity mismatch of China's real enterprises. Using sample data of Chinese A-share listed companies from 2013 to 2020, this article empirically examines the impact of financialization on the investment and financing maturity structure from a new perspective of asset classification by using a fixed-effect model, and explores the mechanism of the financial regulatory environment's moderating effect on the relationship between the two mentioned above. The study shows that: there is an inverted U-shaped nonlinear relationship between the financialization of investment income and fixed income and “maturity mismatch “. The term mismatch of investment and financing increases with the degree of financialization, after reaching the critical point, it eases with the deepening of financialization. However, the specific point of view is different. In the sample interval, the investment income financialization exacerbates the investment and financing maturity mismatch more obviously; the fixed income financialization inhibits the investment and financing maturity mismatch more obviously. Under the different perspectives of the firms' ownership nature, financing constraints, and principal-agent problems, there are differences in the impact of firms' allocation of different types of financial assets on the investment and financing term structure. In addition, the regulatory effect of financial supervision weakens the inverted U-shaped relationship of investment income financialization with investment and financing maturity mismatch; it enhances the inverted U-shaped relationship between fixed income financialization and investment and financing maturity mismatch. In general, financial supervision has had a significant positive effect on investment and financing maturity mismatches. The findings have important policy implications in terms of corporate real investment, financial market development, and financial regulation, which can help promote China's economic development and stability.

## 1 Introduction

The world is now experiencing a major change that has not been seen in a century, and China's high-quality economic development is facing structural transformation and adjustment. In the context of China's proposal to “accelerate the construction of a new development pattern”, a series of outstanding problems, such as high local government debt, pressure on the survival and development of real enterprises, and the intensification of structural contradictions in the real economy, need to be resolved urgently. On the one hand, the return on investment in traditional industries is sluggish, and the intrinsic incentive of fixed-asset investment for the real economy is on a downward trend; on the other hand, the rapid development of China's financial market is driving the investment yield on financial assets upward. Driven by the profit-seeking motive of capital, more and more real enterprises are participating in financial investment in a variety of forms in order to realize their business objectives and obtain excessive profits. The difference between the rate of return on financial investment and that on industrial investment strengthens the potential incentive for real enterprises to invest in financial assets, leading to a large amount of capital leaving the real economy and flowing into the financial and real estate sectors, creating a trend of “financialization” of real enterprises (hereafter referred to as financialization) [1].

Wind database statistics show that a total of 1305 listed companies in China's A-share market purchased wealth management products in 2021, with subscriptions totaling more than 1.36 trillion yuan, and investment in financial assets has become an important investment decision that has a significant impact on the company's operating performance. Some scholars have argued that under conditions of heightened macroeconomic uncertainty, the holding of financial assets helps to maintain the profitability of the real sector [2], whereas over-financialization tends to lead to the cross-pollination and diffusion of risks between the financial and real sectors, which is not conducive to a positive interaction between the financial sector and the real economy. Therefore, it is necessary to explore the substantial impact of the financialization trend on the high-quality development of the Chinese economy.

Most scholars have explored the impact of financialization on corporate investment [3], corporate innovation [4,5], and corporate performance [1,6], resulting in a large number of useful research results. This paper attempts to explore the relationship between financialization and investment and financing maturity mismatch in the light of the debt problems prevailing in Chinese real firms, with a view to providing ideas for policymakers. Capital mismatch is more common in the Chinese economy. From a corporate perspective, the balance sheets of non-financial listed companies show that short-term liabilities accounted for around 80 % of total liabilities and short-term assets accounted for around 60 % of total assets, with the proportion of short-term liabilities continuing to be significantly higher than the proportion of short-term assets. From a credit perspective, the People's Bank of China released a statistical report on the investment of loans by financial institutions in the third quarter of 2022, showing that the balance of short-term loans and bill financing amounted to 49.94 trillion yuan, with a year-on-year growth rate of 14.5 %; the balance of medium-term and long-term loans amounted to 83.86 trillion yuan, with a year-on-year growth rate of 12.7 %, and the proportion of short-term loans in the total amount of loans to enterprises was still relatively high. The structural problem of the mismatch between the asset and liability maturities is prominent, and the phenomenon of enterprises relying on short-term loans to support long-term investment (hereinafter referred to as the “maturity mismatch”) is obvious. Morris [7] was the first to propose the theory of matching the maturity of assets and liabilities, while the Chinese data clearly violate the maturity matching principle. Under the trend of corporate financialization, this paper focuses on issues related to the impact on the maturity structure of investment and financing: first, does financialization “exacerbate” or “inhibit” the maturity mismatch of funds? Second, does the logic of the relationship between the two differ depending on the type of financial assets held? Third, does the implementation of financial regulatory policies have an impact on the relationship between financialization and investment and financing maturity mismatches, and to what extent?

In view of this, using the data samples of Chinese A-share listed non-financial firms, this paper classifies financialization into investment income financialization (IIF) and fixed income financialization (FIF) based on the differences in the returns of financial assets, and explores the impact of holding different types of financial assets on investment and financing maturity mismatches; and then considers the heterogeneous characteristics of the firms in terms of the ownership nature, financing constraints, and principal-agent problems, explores the different manifestations of the impact of financialization on funding maturity mismatches. The study shows that: there is an inverted U-shaped nonlinear relationship between the financialization of investment income and fixed income and “maturity mismatch”. The term mismatch of investment and financing increases with the degree of financialization, after reaching the critical point, it eases with the deepening of financialization. However, the specific point of view is different. In the sample interval, the investment income financialization exacerbates the investment and financing maturity mismatch more obviously; the fixed income financialization inhibits the investment and financing maturity mismatch more obviously. Heterogeneity analysis shows that: the non-state enterprise's allocation of investment income financial assets is more sensitive to investment and financing maturity mismatch than the state enterprise; the state enterprise's allocation of fixed income financial assets is more sensitive to the impact of investment and financing maturity mismatch than non-state enterprises. Enterprises with high financing constraints and low managerial agency conflict are more sensitive to the impact of the allocation of two types of financial assets on investment and financing maturity mismatch than enterprises with low financing constraints and serious managerial agency conflict.

In addition, considering China's special financial regulatory environment, this paper further explores the moderating role of financial regulatory variables on the relationship between financialization and investment and financing maturity mismatch. The results show that: the regulatory effect of financial supervision weakens the inverted U-shaped relationship between investment income financialization and investment and financing term mismatch; it enhances the inverted U-shaped relationship between fixed income financialization and investment and financing maturity mismatch. In general, financial supervision has had a significant positive effect on investment and financing maturity mismatches.

The contributions of this paper to related research are the following: first, against the backdrop of the complex and changing reality of the global economic development situation, it is particularly important to study the relevant status and problems of emerging market countries. Second, financialization has been widely discussed in existing studies, including objective drivers, subjective motives and economic consequences. This paper takes financialization as an entry point to study the problem of investment and financing maturity mismatch in Chinese enterprises, to further enrich and expand the research on the impact of financialization on the consequences of microeconomic real economy, and to provide a useful supplement to the research on the related fields of financialization. Third, most of the current research on financialization explores its economic consequences in terms of precautionary savings motives and profit-seeking motives, but the conceptual definition of financialization of financial assets measured only from the perspective of investment returns is relatively narrow. In this paper, financial assets measured from the perspective of financial intermediation are taken into account, financial assets are reclassified based on the purpose of obtaining investment income and fixed income, and the dual motives and economic consequences of the two types of financialization are explored to enrich the theoretical explanations of the financialization motives. Fourth, this paper analyzes heterogeneity from three perspectives, namely, the ownership nature, financing constraints and principal-agent problems, and the conclusions obtained help to improve the relevance in the formulation and implementation of relevant policies. Fifth, utilizing the changes in China's financial regulatory environment, it correctly evaluates and examines the role and effects of the reform of China's financial regulatory sector, and the content of the study has certain reference significance in promoting the high-quality development of the real economy through the formulation and improvement of financial regulatory policies.

The remainder of the paper is organized as follows: part II, literature review and hypothesis development; part III, research design, including sample data, model and methodology, variable selection, and data descriptive statistics; part IV, empirical results; part V, further research; and part VI, conclusions and recommendations.

## 2 Literature review and hypothesis development

### 2.1 Literature review

#### 2.1.1 Production and economic consequences of financialization

Financialization has been widely studied and explored by scholars. With the development of global economy and financial deepening, a new mode of corporate capital accumulation has emerged by holding financial assets to obtain investment income [8], which is defined by Aalbers [9] as “the structural transformation of firms due to increasing financial activities and scale”. Davis [10] describes the financialization performance of different types of firms using data from the balance sheets of U.S. non-financial corporations (NFCs), pointing out that changes in cash flow and debt structure are important features of financialization behavior. It has been argued that changes in cash flows affect the speed of adjustment of firms' leverage targets [11], which suggests that financialization has the ability to dynamically adjust the capital structure under the condition that there is a yield spread between the financial and real sectors [12].

The trend of financialization of Chinese firms has become more pronounced in recent years, and Yang et al.'s [13] study of China's commodity futures market provides evidence of “financialization”. Most scholars have explored the reasons for firms' financial asset allocation from external environmental factors such as economic policy uncertainty [14] and internal governance factors such as multiple blockholders [3]. Early studies suggested that firms hold financial assets to avoid risk and reduce cash flow volatility due to their judgment of future economic conditions [15]. In recent years, more and more scholars have pointed out that the enterprise's financialization has profit-making motives and may cause serious financial risks [16].

In the study of the financialization's economic consequences, Tomaskovic-Devey et al. [17] pointed out that the increase of financial investment by non-financial firms will cause a decline in the total economic output. Jiang et al. [3] found that financialization significantly inhibits the Chinese firms' investment and increases the firms' business risks. Jin et al. [18] concluded that the Chinese firms' investment in financial assets significantly reduces the fixed investment rate. Zheng et al. [19] found that financialization produces a crowding-out effect on capital accumulation and leads to alienation of corporate investment behavior. Kliman and Williams [20], on the other hand, pointed out that “financialization” did not inhibit productive investment in the United States, and the decline in the rate of capital accumulation was due to the decline in the rate of investment return on fixed assets of U.S. firms. In addition, the literature also focuses on the phenomenon of real firms acting as financial intermediaries to provide financial support to financially disadvantaged firms [21], which is also the scope of this paper's discussion of financialization.

#### 2.1.2 Factors affecting investment and financing maturity mismatch

As an active or passive investment and financing strategy, “enterprises rely on short-term financing for long-term investment” is the main manifestation of investment and financing term mismatch. Proactive capital misallocation decisions can increase liquidity, ease corporate financing constraints and reduce financing costs [22]; whereas passive maturity mismatch can aggravate the debt pressure of enterprises and raise ongoing business risks [23].

Some scholars, on the basis of MM theory [24], pointed out the difference between equity and debt financing channels in terms of cost in reality, and further extended the discussion on the relationship between debt maturity and the capital cost [25], i.e., firms can take the initiative to adjust their capital structure according to changes in the business conditions and the environmental situation by using short-term debts [26]. Such fund mismatches are more prevalent in the Chinese economy, but they stem more from the special institutional environment. China has an indirect financing system dominated by the banking sector, and in order to reduce the risk of information asymmetry and the probability of firms defaulting on loans [27], commercial banks prefer to issue short-term loans and are relatively unwilling to issue long-term loans [28], resulting in the passive formation of firms with a mismatched capital term structure. Cao et al. [29] find that green credit policy can curb investment and financing maturity mismatch risk by reducing short-term bank credit, thanks to the strong enforcement of policy implementation in China. It has also been argued that China's green credit policy reduces short-term and long-term debt financing by commercial banks to heavily polluting firms [30]. Overall, the discussion around the rationality and necessity of maturity mismatch alone in the Chinese economy is insufficient, and more attention should be paid to the debt risks associated with maturity mismatch.

### 2.2 Hypothesis development

#### 2.2.1 Financialization and investment and financing maturity mismatches

It has been pointed out that there are many objective drivers of financialization, and this paper focuses on the impact of financialization on investment and financing maturity mismatch at the level of subjective motives, and financialization can be summarized into the following three motives.

There is a precautionary saving motive for real firms to hold financial assets [14,31,32], which aims to protect against uncertainty. The precautionary savings theory suggests that firms can increase their financial investments to fulfill the function of liquidity reserves, which can be quickly restored through the sale of financial assets when funds are urgently needed, and can be used to protect against external shocks and avoid a break in the financial chain [33]. Studies in different countries and emerging markets provide ample empirical evidence for the theory [4,[34], [35], [36]]. There is a profit-seeking motive for real firms to hold financial assets. Enterprises always seek to maximize profits, and by obtaining investment returns they can improve the overall profitability of the enterprise and enhance the company's performance; therefore, the allocation between financial and real assets can be regarded as a pure investment portfolio [37]. Studies by Xu and Xuan [38] and Huang et al. [39] argue that the dominant motive for Chinese firms' financialization is profit chasing. There is a financial intermediation motive for real firms to hold financial assets. The theory of “entity intermediary " originates from bank credit discrimination and usually exists in countries with underdeveloped formal financial system. Banks have serious financing discrimination against enterprises with small scale and insufficient mortgage, enterprises that are easy to obtain funds from banks can effectively make up for the funding gap of enterprises with financing disadvantages by engaging in financial intermediary business. Firms with financing advantages have built an intermediary bridge between credit-discriminated firms and “big lender” banks [40].

Based on the above motivations, this paper analyzes the mechanism of the role of investment income financialization (IIF) and fixed income financialization (FIF) in affecting investment and financing maturity mismatch, respectively.

Financialization based on the purpose of obtaining investment income has an impact on investment and financing maturity mismatch mainly through the precautionary saving motive and the profit motive. The reason is that holding investment income financial assets does not have a fixed borrowing and lending period, and can better meet the liquidity function. On the one hand, enterprises can play a preventive savings function through financial investment channels to prepare for better real investment opportunities in the future, which not only alleviates the financing constraints and reduces the short-term financing willingness of enterprises, but also meets the demand for long-term investment by reserving sufficient funds so as to further weaken the investment and financing maturity mismatch phenomenon of enterprises. On the other hand, the investment substitution theory generated by the profit-seeking motive holds that excessive participation in financial investment by enterprises will reduce the funds used for real investment, which will have a “crowding out” and “substitution” effect on real investment [16,19,41,42]. With the help of banks' loose short-term credit policy, it is possible to create conditions for enterprises to use short-term loans to make up for the funding gap of long-term investment, thus aggravating the problem of capital maturity mismatch. According to the principal-agent theory [43], management has the motive of using financial asset allocation for personal gain, which may exacerbate the “crowding out” effect of real investment and continue to worsen the investment and financing term mismatch. In addition, Leary and Roberts [44] show that there is a herd effect in leverage and debt maturity decisions. There is a convergence of managers' preferences for short-term maturity structures.

In summary, this paper argues that the dual nature of the motivation for financialization can lead to an unclear effect of financialization on investment and financing term structure. Therefore, the following hypothesis is proposed:

H1a Investment income financialization (IIF) can improve corporate investment and financing term mismatch.

H1b Investment income financialization (IIF) can exacerbate corporate investment and financing term mismatch.

H1c The effect of investment income financialization (IIF) on investment and financing term structure may have non-linear changes.

Financialization, which is based on the purpose of obtaining fixed income, mainly affects investment and financing maturity mismatch through financial intermediation and profit-seeking motives. Enterprises can invest their low-cost funds in the shadow credit system through entrusted loans, private lending and other means to obtain spread income for themselves. If large enterprises are to revitalize idle funds and alleviate the financing constraints of other enterprises, FIF can not only obtain stable returns, but also improve the efficiency of capital utilization. This kind of intermediary activity requires regular debt repayment, and in order to reduce the pressure and risk of debt repayment, the capital provider will pay more attention to the matching of the maturity structure, which will help to alleviate the problem of capital maturity mismatch. If large enterprises are still motivated by profit-seeking, too much capital invested in the shadow banking system will crowd out real investment, jeopardize real production and operation, and exacerbate the risk of capital mismatch. In addition, in the context of soft budget constraints, large enterprises will be subject to lower supervision and constraints, and they will be able to flexibly utilize credit funds and thus have a greater ability to play the role of alternative financing, and excessive borrowing and lending will further exacerbate the maturity mismatch problem.

In summary, this paper proposes the following hypothesis:

H2a Fixed income financialization (FIF) can improve corporate investment and financing term mismatch.

H2b Fixed income financialization (FIF) can exacerbate corporate investment and financing term mismatch.

H2c The effect of fixed income financialization (FIF) on investment and financing term structure may have non-linear changes.

**Fig. 1:**
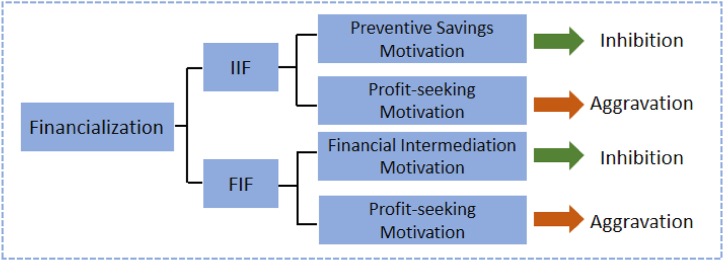
Flowchart of the theoretical framework.

The flowchart of the theoretical framework of this article is illustrated in Fig. 1.

#### 2.2.2 Financialization, financial regulation and investment and financing maturity mismatches

As Cao et al. [29], Peng et al. [30] and Li et al. [45] have shown, in order to realize the goal of green development, the government, as a representative of public interest, adopts green credit policies and environmental regulations to motivate enterprises to take the initiative in eliminating outdated production capacity, accelerating the green transformation, and realizing the economic sustainable development. Financial regulation can play a similar role in solving speculative arbitrage and inefficiency problems in the market and maintaining the normal order of a country's economy and finance. At present, the impact of financial regulation on corporate investment and financing term mismatch remains to be studied. On the one hand, based on the fund supply perspective, the construction of the financial regulatory system may break the original balance of fund supply and demand, and financial institutions, in order to implement the requirements of the relevant risk indicators, may reduce their appetite for risk, reduce the scale of medium-term and long-term loans [46], and control the moral hazard of the enterprise with the help of more short-term credit contracts [47], which exacerbates the degree of enterprise capital maturity mismatch; On the other hand, based on the fund demand perspective, the financial regulatory policy is aimed at eliminating regulatory gaps and guiding the return of finance to its origin. By curbing illegal and irregular operations, it can enhance the investment expectations of market entities and enterprises' own risk awareness, guide enterprises to actively adjust their capital flows, increase the medium-term and long-term capital allocation, optimize the capital maturity structure, and improve the ability of enterprises to cope with risk impacts with their main business operations.

Due to the enterprise's limited use and allocation of endogenous and exogenous funds, once financialization is excessive, it will directly affect the enterprise's production and operation and strategic layout, which in turn affects the enterprise's investment and financing term structure. In this process, the difference in the external financial regulatory environment is crucial, the stricter the regulatory requirements, the more prudent the financialization behavior, the more it can reduce the sensitivity to changes in the fund maturity structure, resulting in a different impact of financialization on the investment and financing term mismatch. On the one hand, the strengthening of financial regulation may further deteriorate the financing conditions of enterprises that do not have “hard assets” [48], increasing the cost of enterprise financing and survival pressure, at which time enterprises may choose to increase liquidity, further aggravating the investment and financing term mismatch. On the other hand, financial regulation can enhance the operational efficiency of the financial market [49], such as through the suppression of multi-layer nesting to achieve penetrating regulation, and form an effective supervision of financial investment activities, which not only prevents the funds from idling in the financial system, but also guides the accumulation of social funds to the real economy, thus weakening the willingness of enterprises to make financial investments and correcting the investment and financing term mismatch. In addition, more open and diversified financing channels can improve the long-term debt financing and equity financing capacity of enterprises and further optimize the fund term structure.

In summary, the effect of the role played by financial regulation may be different under different influence paths. Therefore, this paper proposes the following hypothesis:

H3a Financial regulation has a positive moderating effect in the impact of financialization on corporate investment and financing term mismatch.

H3b Financial regulation has a negative moderating effect in the impact of financialization on corporate investment and financing term mismatch.

## 3 Research design

### 3.1 Data and sample selection

There are great differences between developed and developing countries in terms of financial systems, financial market structures and the objectives and means of financial regulation. As the largest developing country, it is of practical significance to study the current situation and problems in China. Considering that the financial data of listed companies are relatively sound and can intuitively reflect the trend of financialization and the debt maturity structure of enterprises, this paper, like most scholars [1,6,[50], [51], [52]], chooses China's A-share listed companies as the object of study.

The data of Chinese A-share listed companies from 2013 to 2020 are selected as samples. The data source is China Stock Market and Accounting Research (CSMAR) database. The following criteria are used to filter the samples: firstly, the samples of the companies that are in the financial industry in China Securities Regulatory Commission (CSRC) 2012 version of the industry classification are excluded; secondly, the samples of ST, *ST are excluded; and lastly, the samples of the insolvent as well as the missing data are excluded. All continuous variables are subjected to 1 % and 99 % reduction of tails. The paper finally obtains a sample of 17931 unbalanced observations.

### 3.2 Model and method

This paper uses a two-way fixed effects model to test the impact of investment income financialization (IIF) and fixed income financialization (FIF) on investment and financing maturity mismatch. To test the hypotheses, this paper constructs the following linear regression model (1) and nonlinear regression model (2):

(1) *SFLI*_*i,t*_ = *α*_*0*_ + *α*_*1*_*IIF/FIF*_*i,t*_ + *∑CONTROL*_*i,t*_ + *∑IND*_*j*_ + *∑YEAR*_*t*_ + *ε*_*i,t*_

(2) *SFLI*_*i,t*_ = *β*_*0*_ + *β*_*1*_*IIF/FIF*_*i,t*_ + *β*_*2*_*IIF*^*2*^*/FIF*^*2*^_*i,t*_ + *∑CONTROL*_*i,t*_ + *∑IND*_*j*_ + *∑YEAR*_*t*_ + *ε*_*i,t*_

Among equations (1), (2)), i denotes individual firms, t denotes year, SFLI is an indicator of the dependent variable “maturity mismatch ", while IIF and FIF are indicators of the independent variable “financialization”, respectively. ∑ CONTROL represents all control variables at the enterprise level, ∑ IND is the industry fixed effect, ∑ YEAR is the year fixed effect, and ε represents the random disturbance term. The α_0_ in Model (1) and the β_0_ in Model (2) represent the average of all individual effects, respectively. The α_1_ in model (1) and the β_1_ and β_2_ in model (2) describe the linear and nonlinear relationship between financialization and investment and financing maturity mismatch respectively.

Referring to Haans et al. [53], this paper uses the interaction term of financialization and financial regulation to test the moderating effect and constructs the following regression models (3) and (4):

(3) *SFLI*_*i,t*_ = *μ*_*0*_ + *μ*_*1*_*X*_*i,t*_ + *μ*_*2*_*X*_i,t_* POST_*t*_ + *μ*_*3*_*POST*_*t*_ + *∑CONTROL*_*i,t*_ + *∑IND*_*j*_ + *∑YEAR*_*t*_ + *ε*_*i,t*_

(4) *SFLI*_*i,t*_ = *γ*_*0*_ + *γ*_*1*_*X*_*i,t*_ + *γ*_*2*_*X*^*2*^_*i,t*_ + *γ*_*3*_*X*_i,t_* POST_*t*_ + *γ*_*4*_*X*^*2*^_i,t_* POST_*t*_ + *γ*_*5*_*POST*_*t*_ + *∑CONTROL*_*i,t*_ + *∑IND*_*j*_ + *∑YEAR*_*t*_ + *ε*_*i,t*_

In equations (3), (4)) X stands for IIF and FIF, respectively. If the regression indicates that there is only a linear relationship between financialization and investment and financing maturity mismatch, then the model (3) is used to test by introducing the cross-multiplier terms of financialization and policy year dummy variables and if the coefficients of the cross-multiplier terms are significant, it indicates that there is a moderating effect on the above relationship. If the regression indicates that there is a non-linear relationship, the test is conducted using model (4) to examine whether the coefficients of the cross-multiplier terms, γ3 and γ4, are significant.

### 3.3 Measurement of variables

#### 3.3.1 Dependent variable

The dependent variable is the “maturity mismatch” indicator (SFLI), which is measured by Frank and Goyal [54]. The main idea is to utilize the funds expended by the enterprise on long-term investments minus the funds obtained through non-short-term financing. The difference being the enterprise's portion of long-term investments financed through short-term debt (see Table 1 for specific settings). The value is smoothed with the previous total assets, and finally the “maturity mismatch” proxy variable SFLI is obtained. The larger the value of the variable, the more serious the “maturity mismatch” of the enterprise. In addition, the dummy variable DUM_SFLI of “maturity mismatch” is set. If the enterprise has “maturity mismatch”, SFLI >0, DUM_SFLI = 1; if there is no “maturity mismatch”, SFLI <0, then DUM_SFLI = 0.

**Table 1:** Variable definition.

Variable	Variable symbol	Definition
Maturity mismatch	SFLI	Balance sheet and cash-flow statement calculation. Cash expenditure on investment activities such as purchase and construction of fixed assets - (long-term loan increase in the current period + increase in equity in the current period + net cash flow from operating activities + cash inflow from selling fixed assets), use of prior year's total assets to remove scale effects
Dummy variable	DUM_SFLI	SFLI >0, DUM_SFLI = 1; SFLI <0, DUM_SFLI = 0.
Financialization	IIF	Balance sheet calculation. Holding investment income financial assets (six accounting subjects)/total assets in the current period
	FIF	Balance sheet calculation. Holding fixed income financial assets (four accounting subjects)/total assets in the current period
Financial regulation	POST	Indicates the year of implementation of the “New Regulation on Capital Management”, with POST = 1 for 2018 and later years, and POST = 0 for pre-2018 years.
Company size	Size	Ln(total assets)
Asset-liability ratio	Lev	Total liabilities/total assets
Tobin Q	Q	Market value/total assets
Company growth	Growth	Current operating income/previous operating income
Company profitability	Roa	Net profit/total assets
Ongoing operation	Age	Ln(operating year - company listing year + 1)
Ownership structure	Top1	The number of shares held by the largest shareholder of the company/the total number of shares
Whether the company's executives have financial background	Executive	Some of the company executives in each year have financial background, Executive = 1; otherwise Executive = 0
Ownership	Property	According to the actual control nature judgment. State enterprises, Property = 1; non-state enterprises, Property = 0

#### 3.3.2 Independent variable

The independent variable is the “financialization” index (IIF/FIF), referring to the practice of Demir [16], from the new perspective of financial asset classification, financial assets are divided into investment income financial assets and fixed income financial assets. Measuring the level of financialization using the sum of relevant accounting entries, the six accounting subjects of “held-for-trading financial assets “, “derivative financial assets “, “buying back the sale of financial assets “, “available-for-sale financial assets “, “held-to-maturity investment” and “investment real estate” are divided into investment income financial assets; the four accounting subjects of “other current assets “, “non-current assets due in one year “, “other non-current assets” and “other receivables” are divided into fixed income financial assets. Dividing financial assets by total assets to obtain the proxy variable of financialization, IIF is investment income financialization, FIF is fixed income financialization.

#### 3.3.3 Moderator variable

This paper uses relevant policy guidelines that can effectively prevent financial risks and regulate the operation of financial institutions to reflect the environmental changes in financial supervision. The “New Asset Management Regulations”, which is the first unified regulatory policy to be introduced, facilitates the research of financial regulation's impact on micro firms. The regulatory object of this programmatic document covers all enterprises and financial institutions, so all samples in this paper are affected by the policy. POST is used to represent the year in which the " New Asset Management Regulations " are implemented, in 2018 and later years, it is set to POST = 1, and before 2018, it is set to POST = 0 to study the regulatory role of regulatory policy changes in the impact of financialization on investment and financing maturity mismatch.

#### 3.3.4 Control variable

Economic uncertainty and intensified external competition have led to changes in the enterprise's business decisions and its increasing sensitivity to capital maturity mismatches. In addition to the impact of the investment and financing environment, policy changes and other factors, differences in corporate organizational structure, management model, performance, etc. may have an impact on the way in which the enterprise's capital is operated [55]. Based on the existing literature [1,[4], [5], [6]], the article selects company size, asset-liability ratio, tobin Q, company growth, company profitability, ongoing operation, ownership structure, whether the company's executives have financial background and ownership as control variables.

Company Size (Size): Under economies of scale, large firms have cost and financing advantages, and they are more flexible in restructuring their capital and debt.

Asset-liability Ratio (Lev): This indicator reflects the financing structure of the enterprise in the process of production and operation, if the solvency of the enterprise is guaranteed, the funds financed to the enterprise will not be too risky.

Tobin Q (Q): Tobin Q measures the stock market's future expectations of firms [1], and its value has a significant impact on firms' investment decisions.

Company Growth (Growth): Companies with a more stable trend in operating income growth rate can enhance the judgment of external investors on the company's operating ability.

Company Profitability (Roa): Effectively measure the efficiency of asset utilization and profitability of the enterprise.

Ongoing Operation (Age): The longer the effective existence of a listed company, the higher its credibility and social recognition tend to be, which is crucial for attracting investors.

Ownership Structure (Top1): Shareholder status allows for the assertion of rights over the company, and different shareholding structures determine different corporate governance structures, which ultimately affect corporate behavior and decision-making.

Whether the Company's Executives Have Financial Background (Executive): Management with a financial background has appropriate access to financial market information and resources and is able to control corporate funds, their diversified investment decisions will affect the entity's business and debt maturity structure [56].

Ownership (Property): Enterprises with different nature of ownership have their own characteristics and strengths, which may manifest themselves differently in terms of the maturity structure of funds.

The above control variables are the key variables affecting corporate investment and financing decisions. See Table 1 for specific settings.

### 3.4 Data descriptive statistics

Table 2 lists the main variables' descriptive statistics. The results show that the proxy variable SFLI has a mean value of −0.124, the minimum and maximum values are −1.657 and 0.271 respectively, which indicates that the investment and financing term structure varies greatly among various companies. The mean value of the dummy variable DUM_SFLI is 0.221, reflecting the existence of the phenomenon of " maturity mismatch " in almost a quarter of the company samples. The mean values of financialization proxy variables IIF and FIF are 0.039 and 0.067 respectively, that is, the two types of financial assets account for an average of 3.9 % and 6.7 % of firms' total assets, respectively, suggesting that fixed income financialization is generally higher than that of investment income in the sample. Other company-level variable indicators are no longer repeated, which is generally similar to existing studies.

**Table 2:** Data descriptive statistics.

Variable	Obs	Mean	SD	Min	P25	P50	P75	Max
SFLI	17931	−0.124	0.250	−1.657	−0.164	−0.078	−0.009	0.271
DUM_SFLI	17931	0.221	0.415	0.000	0.000	0.000	0.000	1.000
IIF	17931	0.039	0.071	0.000	0.001	0.010	0.042	0.409
FIF	17931	0.067	0.076	0.001	0.019	0.040	0.084	0.409
Size	17931	22.390	1.322	15.580	21.470	22.210	23.120	28.540
Lev	17931	0.431	0.202	0.060	0.270	0.422	0.582	0.882
Q	17931	2.440	1.760	0.832	1.295	1.866	2.900	10.600
Growth	17931	1.160	0.409	0.439	0.969	1.094	1.248	3.607
Roa	17931	0.034	0.061	−0.265	0.013	0.034	0.063	0.186
Age	17931	2.308	0.710	0.000	1.792	2.398	2.944	3.434
Top1	17931	34.450	14.800	2.197	23.030	32.260	44.310	89.990
Executive	17931	0.226	0.418	0.000	0.000	0.000	0.000	1.000
Property	17931	0.382	0.486	0.000	0.000	0.000	1.000	1.000

## 4 Empirical analysis

### 4.1 Basic regression results and discussion

Table 3 is the basic regression result. Columns (1) and (2) present the results of financialization (IIF) in Model 1 and Model 2, respectively. The results show that there is a significant linear positive correlation between IIF and SFLI (α_1_ = 0.082, p < 0.05), the phenomenon that financialization aggravates investment and financing maturity mismatch is obvious. After adding the quadratic term, IIF shows a significant inverted U-shaped relationship with SFLI (β_1_ = 0.259, p < 0.05, β_2_ = −0.593, p < 0.1), that is, investment and financing maturity mismatch intensify with the degree of financialization, and then decrease with the deepening of financialization after reaching the critical point, which verifies the hypothesis H1c in this paper. Enterprises based on profit-seeking motives by holding a certain amount of financial assets to improve the overall rate of return on investment, forming an obvious crowding-out effect on the entity investment in the short term, exacerbating the capital maturity mismatch; while holding too many financial assets on behalf of the enterprise has sufficient cash flow and available funds, the liquidity advantage makes the enterprise can flexibly cope with the market changes and investment opportunities, to meet the various short-term and long-term operational needs, which improves the degree of investment and financing maturity mismatch of the enterprise.

**Table 3:** The impact of financialization on investment and financing maturity mismatch.

Dependent variable	SFLI			
	(1)	(2)	(3)	(4)
IIF	0.082** (2.32)	0.259** (2.01)		
Square_IIF		−0.593* (−1.80)		
FIF			−0.081*** (−5.71)	0.193*** (4.42)
Square_FIF				−0.870*** (−5.84)
Size	−0.160*** (−3.34)	−0.160*** (−3.35)	−0.160*** (−3.34)	−0.162*** (−3.35)
Lev	0.366*** (2.95)	0.365*** (2.95)	0.361*** (2.94)	0.361*** (2.93)
Q	−0.012*** (−4.00)	−0.011*** (−3.98)	−0.011*** (−4.03)	−0.011*** (−4.04)
Growth	−0.221*** (−20.96)	−0.221*** (−20.95)	−0.222*** (−21.13)	−0.222*** (−21.10)
Roa	−0.680*** (−6.36)	−0.681*** (−6.39)	−0.685*** (−6.45)	−0.680*** (−6.40)
Age	0.007 (0.18)	0.005 (0.12)	0.008 (0.21)	0.009 (0.24)
Top1	0.00067*** (3.35)	0.00067*** (3.26)	0.00068*** (3.41)	0.00070*** (3.48)
Property	−0.004 (−0.78)	−0.004 (−0.64)	−0.004 (−0.77)	−0.005 (−1.00)
Executive	0.010** (1.96)	0.010** (1.97)	0.010** (1.98)	0.010** (1.99)
_cons	3.279*** (3.50)	3.274*** (3.50)	3.298*** (3.50)	3.313*** (3.51)
Year effect	Yes	Yes	Yes	Yes
Industry effect	Yes	Yes	Yes	Yes
Observations	17931	17931	17931	17931
adj.R^2^	0.3299	0.3301	0.3301	0.3307

Notes: In this article, *p < 0.1, * *p < 0.05, * * *p < 0.01; the t statistic is in brackets; the following table is the same.

In columns (3) and (4), the results of financialization (FIF) in model 1 and model 2 are reported respectively. Slightly different from the above conclusions, FIF is significantly and linearly negatively correlated with SFLI (α_1_ = −0.081, p < 0.01), suggesting that the effect of financialization in suppressing investment and financing maturity mismatch is more significant. After adding the quadratic term, the regression results still support the inverted U-shaped relation of FIF with SFLI (β_1_ = 0.193, p < 0.01, β_2_ = −0.870, p < 0.01), which verifies the hypothesis H2c. Firms engaging in financial intermediation by holding fixed-income financial assets exacerbate the investment and financing maturity mismatch at the initial stage, but significantly improve it in the long run. The reason is that the allocation of this type of financial assets belongs to the profit model of recovering principal and interest at maturity, which is lower risk and has a low spread return compared to the investment return in the stock market. In order to maximize the use of idle funds, enterprises will combine their own needs and risk preferences, rationally arrange the term of idle funds, reduce the risk of repayment of borrowed funds, while optimizing the enterprise target capital structure adjustment.

In addition, the larger the scale, the lower the asset-liability ratio, the better the investment opportunities, the higher the growth, the stronger the profitability, the lower the degree of investment and financing maturity mismatch; enterprises with concentrated equity and executives with financial background have a higher degree of investment and financing term mismatch, indicating the impact of corporate agency problems on investment and financing decisions.

### 4.2 Robustness checks

#### 4.2.1 Instrumental variable method

The endogenous problem is considered. For reducing the endogeneity problem due to the omission of independent variables, this article adds more firm-level control variables to the model setup, while utilizing a two-way fixed-effects model for the regression. In order to eliminate the missing variables and measurement errors caused by unobservable factors, as well as the reverse causality problem, the article uses the instrumental variable method to estimate.

For IIF, the financialization index IV1_IIF excluding “investment real estate” and its square term Square_IV1_IIF and “net investment real estate” IV2_IIF are used as instrumental variables. For FIF, the lagged financialization index FIF_t-1_ and its square term Square_FIF_t-1_ are used as instrumental variables. The selection of instrumental variables meets the basic assumptions, the two-stage estimation results are shown in Table 4. Column (3) shows the second-stage regression results of IIF affecting firms' investment and financing maturity mismatch, and the coefficients of IIF and Square_IIF adjusted by instrumental variables are 0.421 and −0.950 respectively, which are both significant at the 1 % level. Column (6) shows the results of the second-stage regression of FIF affecting firms' investment and financing maturity mismatches, and the coefficients of FIF and Square_FIF adjusted for instrumental variables are 2.138 and −5.905, respectively, which are both significant at the 1 % level, indicating that there is a notable nonlinear effect of both investment income and fixed income financialization on investment and financing maturity mismatches. The two-stage least-squares estimation results are basically in line with those of the basic regression.

**Table 4:** Robustness Check_ Instrumental variable method.

	First stage		Second stage	First stage		Second stage
Dependent variable	IIF	Square_IIF	SFLI	FIF	Square_FIF	SFLI
	(1)	(2)	(3)	(4)	(5)	(6)
IV1_IIF	0.894*** (101.27)	−0.042*** (−11.63)				
Square_IV1_IIF	0.607*** (17.12)	1.459*** (99.62)				
IV2_IIF	0.004*** (72.69)	0.001*** (55.55)				
IIF			0.421*** (4.14)			
Square_IIF			−0.950*** (−2.95)			
FIF_t-1_				0.270*** (12.06)	0.033*** (4.20)	
Square_FIF_t-1_				0.022 (0.34)	0.165*** (7.39)	
FIF						2.138*** (4.62)
Square_FIF						−5.905*** (−4.06)
_cons	Yes	Yes	Yes	Yes	Yes	Yes
Control	Yes	Yes	Yes	Yes	Yes	Yes
Year effect	Yes	Yes	Yes	Yes	Yes	Yes
Industry effect	Yes	Yes	Yes	Yes	Yes	Yes
Observations	17924	17924	17924	14745	14745	14745

#### 4.2.2 Replace the dependent variable

This article replaces the measurement indicators of the dependent variables to re-regression. The following indicators are constructed to measure investment and financing maturity mismatch: SFLI_SUB = (long-term assets - long-term liabilities - shareholders ' equity)/long-term assets, which means the proportion of long-term assets supported by short-term financing. If the term structure of funds matches, the index is negative; the more serious the maturity mismatch, the greater the SFLI_SUB value. Based on the indicator's characteristics, this article performs a 2.5 % left-handed one-sided shrinkage of the original data. Columns (2) and (4) verify that IIF and FIF have an inverted U-shaped relationship with “maturity mismatch” (see Table 5), with the coefficients of the quadratic terms are −5.190 and −6.614, which are significantly negative at the 1 % level, while the model has an adjusted R^2^ of 0.77, which is a better fit. The estimation results using this index are basically consistent with the previous ones.

**Table 5:** Robustness Check_ Replace the dependent variable.

Dependent variable	SFLI_SUB			
	(1)	(2)	(3)	(4)
IIF	0.840 (1.38)	2.384*** (2.61)		
Square_IIF		−5.190*** (−4.68)		
FIF			−0.947*** (−7.48)	1.142*** (3.18)
Square_FIF				−6.614*** (−5.60)
_cons	−2.858*** (−6.07)	−2.901*** (−6.09)	−2.648*** (−6.47)	−2.532*** (−6.25)
Control	Yes	Yes	Yes	Yes
Year effect	Yes	Yes	Yes	Yes
Industry effect	Yes	Yes	Yes	Yes
Observations	17930	17930	17930	17930
adj.R^2^	0.7711	0.7719	0.7722	0.7738

#### 4.2.3 Sample of manufacturing enterprises

As an important mainstay of China's national economy, manufacturing enterprises have the largest number of listed companies, and their investment and financing term structure best reflects the level of China's microeconomic development and the degree of perfection of its market system. For the sake of robustness, the definition of entity enterprises is narrowed to a narrow category, including only manufacturing enterprises. The sample is selected based on the 2012 version of the CSRC's classification standard for manufacturing industries, and 11384 observed value samples were obtained. The main conclusions (see Table 6) in the manufacturing sample are still valid, the coefficient estimate of the quadratic term Square_IIF in column (2) is −0.531, which is significant at the 10 % level, and the coefficient estimate of the quadratic term Square_FIF in column (4) is −0.887, which is significant at the 1 % level, there is a more significant non-linear effect of investment income and fixed income financialization on investment and financing maturity mismatch. After reducing the sample size, it still has sufficient explanatory power for the original hypothesis.

**Table 6:** Robustness Check_ Sample of manufacturing enterprises.

Dependent variable	SFLI			
	(1)	(2)	(3)	(4)
IIF	0.099** (2.39)	0.247** (2.44)		
Square_IIF		−0.531* (−1.89)		
FIF			−0.116*** (−2.81)	0.162* (1.66)
Square_FIF				−0.887*** (−2.83)
_cons	3.922*** (17.06)	3.919*** (17.06)	3.923*** (17.05)	3.932*** (17.13)
Control	Yes	Yes	Yes	Yes
Year effect	Yes	Yes	Yes	Yes
Observations	11384	11384	11384	11384
adj.R^2^	0.3556	0.3557	0.3560	0.3567

### 4.3 Heterogeneity results

#### 4.3.1 Ownership nature

According to the different ownership of enterprises, the samples are divided into state enterprises and non-state enterprises, the impact of IIF and FIF on investment and financing term structure is explored respectively (see Table 7). Columns (1) and (3) report the nonlinear relationship between IIF and SFLI. When other variables are constant, the inverted U-shaped curve in the non-state enterprise sample is steeper than that in the state enterprise sample, that is, compared with state enterprises, non-state enterprises do not have implicit guarantees and are more susceptible to the impact of macro-environmental uncertainty and other factors, and thus face greater operational risks and probability of bankruptcy, the stronger performance pressure in this process will prompt enterprises to improve overall economic efficiency through the allocation of more investment income financial assets, to maintain their industry competitiveness in the market, and the adjustment of the corporate debt maturity structure will also generate greater volatility.

**Table 7:** Heterogeneity analysis of ownership nature.

	State enterprise		Non-state enterprise	
Dependent variable	SFLI			
	(1)	(2)	(3)	(4)
IIF	0.191* (1.88)		0.284* (1.95)	
Square_IIF	−0.585* (−1.90)		−0.708** (−2.02)	
FIF		0.281*** (2.96)		0.126** (2.14)
Square_FIF		−1.040*** (−3.55)		−0.680*** (−3.35)
Size	−0.137*** (−3.40)	−0.139*** (−3.45)	−0.176*** (−3.27)	−0.178*** (−3.26)
Lev	0.309** (2.45)	0.309** (2.49)	0.397*** (3.15)	0.392*** (3.13)
Q	−0.001 (−1.63)	−0.001* (−1.80)	−0.011*** (−3.08)	−0.011*** (−3.14)
Growth	−0.236*** (−25.54)	−0.236*** (−25.64)	−0.209*** (−13.70)	−0.210*** (−13.86)
Roa	−0.697*** (−7.26)	−0.704*** (−7.50)	−0.665*** (−5.82)	−0.663*** (−5.70)
Age	−0.053*** (−2.99)	−0.050*** (−2.87)	0.019 (0.46)	0.022 (0.53)
Top1	−0.00107 (−1.31)	−0.00103 (−1.28)	0.00210*** (4.44)	0.00212*** (4.34)
Executive	−0.001 (−0.17)	−0.001 (−0.19)	0.011 (1.40)	0.012 (1.40)
_cons	3.370*** (3.88)	3.395*** (3.91)	3.091*** (2.65)	3.148*** (2.65)
Year effect	Yes	Yes	Yes	Yes
Industry effect	Yes	Yes	Yes	Yes
Observations	6847	6847	11084	11084
adj.R^2^	0.3698	0.3705	0.3292	0.3296

Columns (2) and (4) report the nonlinear relationship between FIF and SFLI. When other variables are constant, the inverted U-shaped curve in the state enterprise sample is steeper than that in the non-state enterprise sample, that is, China's state enterprises may undertake policy tasks in their operations with the aim of stabilizing the economy, safeguarding people's livelihoods and resolving employment, so they can receive financial support from the government even if they are in poor operating conditions, a process in which enterprises tend to over finance and assume the role of financial intermediaries, utilizing fixed-income financial assets to seek spread returns and play a stronger alternative financing function, which in turn affects the corporate investment and financing maturity mismatch.

In addition, in the sample of state enterprises, the longer the duration of continuous operation, the lower the degree of maturity mismatch; in the sample of non-state enterprises, the concentration of shareholders ' equity aggravates investment and financing maturity mismatch.

#### 4.3.2 Financing constraint

Considering that the SA index does not contain endogenous statistical variables, this article uses the SA index to serve as a proxy variable for corporate financing constraints, which is negative, and the larger the absolute value, the higher the financing constraints. The samples are divided into high financing constraint enterprise samples and low financing constraint enterprise samples by sample median. The results (see Table 8) suggest that the inverted U-shaped curve between IIF, FIF and SFLI is steeper in the sample of high finance constrained firms than in the sample of low finance constrained firms. That is to say, the investment and financing maturity mismatch phenomenon of Chinese enterprises can be regarded as a kind of performance under the background of financial inhibition, which is the passive capital allocation strategy of enterprises, and in the future, with the help of various measures to improve the defects of the financial market system, it can optimize the investment and financing maturity structure, and reduce the business risks of enterprises.

**Table 8:** Heterogeneity analysis of financing constraint.

	High financing constraints		Low financing constraints	
Dependent variable	SFLI			
	(1)	(2)	(3)	(4)
IIF	0.321* (1.74)		0.282* (1.94)	
Square_IIF	−0.837* (−1.79)		−0.652** (−1.96)	
FIF		0.383*** (4.80)		0.137*** (2.91)
Square_FIF		−1.304*** (−6.34)		−0.737*** (−5.07)
Size	−0.161*** (−3.94)	−0.164*** (−3.95)	−0.216*** (−4.19)	−0.217*** (−4.21)
Lev	0.404*** (3.65)	0.402*** (3.68)	0.546*** (3.75)	0.538*** (3.75)
Q	−0.011*** (−4.13)	−0.011*** (−4.21)	−0.009* (−1.95)	−0.009** (−2.03)
Growth	−0.223*** (−12.37)	−0.223*** (−12.46)	−0.206*** (−21.50)	−0.207*** (−21.93)
Roa	−0.610*** (−5.99)	−0.609*** (−5.96)	−0.555*** (−4.69)	−0.558*** (−4.81)
Age	0.045 (0.92)	0.055 (1.12)	0.037 (0.85)	0.040 (0.91)
Top1	0.00008 (0.09)	0.00018 (0.21)	0.00116*** (4.40)	0.00115*** (4.23)
Property	−0.007 (−1.41)	−0.009** (−2.26)	−0.007 (−0.49)	−0.010 (−0.66)
Executive	0.009 (1.16)	0.009 (1.18)	0.016** (2.15)	0.017** (2.26)
_cons	3.397*** (4.11)	3.495*** (4.19)	4.737*** (4.35)	4.761*** (4.35)
Year effect	Yes	Yes	Yes	Yes
Industry effect	Yes	Yes	Yes	Yes
Observations	8966	8966	8965	8965
adj.R^2^	0.3428	0.3435	0.3458	0.3464

In addition, the effects of concentrated equity structure and executive financial background on investment and financing maturity mismatch are significant among low financing constraint firms.

#### 4.3.3 Manager agency problem

The principal-agent problem can reflect corporate governance levels to a certain extent [57]. If the internal agency conflict is serious, it indicates that the company has problems in the governance structure and mechanism [58]. Generally speaking, the agency problem of controlling shareholders is more hidden, while the agency problem of managers is more obvious. This part selects the agency cost rate as the measurement index of the manager's agency problem. Agency = (management cost + sales cost)/operating income, the more severe the managerial agency problem, the greater the value of the Agency indicator. Through the median of the sample, the sample is divided into the sample of enterprises with serious agency problems and the sample of enterprises with lighter agency problems, reflecting the effect of the level of corporate governance on the main causal relationship (see Table 9). In the sample of enterprises with lighter agency problems, the inverted U-shaped curve between IIF, FIF and SFLI is steeper than that of enterprises with serious agency problems. The possible explanation is that firms with less agency problems, i.e., better corporate governance, are more flexible as they focus more on target capital structure adjustments in their capital allocation strategies and decisions.

**Table 9:** Heterogeneity analysis of manager agency problem.

	Serious agency problem		Lighter agency problem	
Dependent variable	SFLI			
	(1)	(2)	(3)	(4)
IIF	0.282*** (2.60)		0.354*** (2.82)	
Square_IIF	−0.755** (−2.50)		−0.887** (−2.36)	
FIF		0.205* (1.79)		0.280** (2.11)
Square_FIF		−0.738** (−2.19)		−1.174** (−2.52)
Size	−0.193*** (−15.33)	−0.195*** (−15.47)	−0.150*** (−12.00)	−0.152*** (−12.18)
Lev	0.391*** (9.29)	0.389*** (9.27)	0.415*** (9.70)	0.410*** (9.52)
Q	−0.010*** (−3.55)	−0.010*** (−3.62)	−0.014*** (−3.25)	−0.014*** (−3.20)
Growth	−0.221*** (−12.92)	−0.222*** (−12.91)	−0.237*** (−14.95)	−0.237*** (−14.95)
Roa	−0.642*** (−11.28)	−0.638*** (−11.17)	−0.660*** (−9.87)	−0.669*** (−9.91)
Age	0.036 (1.56)	0.041* (1.82)	−0.027 (−1.41)	−0.021 (−1.09)
Top1	0.00164** (2.47)	0.00165** (2.50)	0.00054 (0.81)	0.00058 (0.88)
Property	0.036* (1.75)	0.033 (1.61)	−0.034 (−1.54)	−0.034 (−1.53)
Executive	0.011 (1.21)	0.011 (1.18)	0.013 (1.45)	0.014 (1.53)
_cons	3.404*** (13.03)	3.443*** (13.18)	3.609*** (12.59)	3.636*** (12.73)
Year effect	Yes	Yes	Yes	Yes
Industry effect	Yes	Yes	Yes	Yes
Observations	8965	8965	8966	8966
adj.R^2^	0.3396	0.3398	0.3826	0.3832

In addition, in the sample of enterprises with serious agency problems, the concentration of ownership structure has a significant impact on the maturity mismatch.

## 5 Further research: the effect of financialization under financial supervision

The trend of real business financialization has caused obvious impacts on the real economy and the financial system, and has also posed new challenges to traditional financial regulation. In April 2018, the People's Bank of China and other departments jointly issued the “New Asset Management Regulations”, which gave clear definitions and standards in financial institutions, investors, asset management business and product innovation. The policy has set the main tone of cracking down on regulatory arbitrage and reconstructing the order of financial markets, which will have an important impact on corporate investment and debt maturity structure adjustment.

Considering the inverted U-shaped relationship of the main causal variables, model 4 was utilized for the test. Table 10 presents the empirical results. According to the conclusion of Haans et al. [53], whether the critical point (“inflection point”) of the inverted U-shaped curve moves ultimately depends on the positive and negative values of γ_1_γ_4_-γ_2_γ_3_. If γ_1_γ_4_-γ_2_γ_3_ > 0, the inflection point will move to the right as the policy occurs, otherwise it will move to the left; testing whether the inverted U-shaped curve becomes flat or steep depends on whether the cross term coefficient γ_4_ of the independent variable quadratic term and financial supervision is significant.

**Table 10:** The moderating effect test of financial regulation.

Dependent variable	SFLI	
	(1)	(2)
IIF	0.369*** (2.79)	
Square_IIF	−0.950*** (−3.22)	
FIF		0.081 (1.25)
Square_FIF		−0.599*** (−2.76)
IIF*POST	−0.204** (−2.46)	
Square_IIF*POST	0.663*** (3.64)	
FIF*POST		0.303** (2.47)
Square_FIF*POST		−0.739* (−1.71)
POST	0.079*** (3.08)	0.065*** (2.96)
_cons	3.276*** (3.49)	3.318*** (3.52)
Control	Yes	Yes
Year effect	Yes	Yes
Industry effect	Yes	Yes
Observations	17931	17931
adj.R^2^	0.3302	0.3309

Roughly speaking, in columns (1) and (2), γ_1_γ_4_-γ_2_γ_3_ > 0, indicating that the critical point of the inverted U-shaped curve of the two types of financial assets has shifted to the right. The key point is that column (1) reports the results of IIF in model 4, the cross-multiplication coefficient γ_4_ is 0.663, which is significant at the level of 1 %. The inverted U-shaped relationship between IIF and SFLI becomes flat, that is, affected by changes in regulatory policies, market resources have been reallocated, the phenomenon of real enterprises chasing financial investment returns in the past has been effectively curbed and restrained, and more funds have been utilized for real project investments, weakening the crowding-out effect and improving the term mismatch of enterprise funds. Column (2) reports the results of FIF in Model 4, the coefficient of cross term γ_4_ is −0.739, which is significant at the level of 10 %. The inverted U-shaped relationship between FIF and SFLI becomes steep, that is, under the policy's influence, financialization behaviors have become more prudent, and firms will hold excessive amounts of underutilized idle funds and invest them in the real economy, thereby optimizing the capital maturity structure.

Overall, financial supervision has had a significant positive effect on investment and financing maturity mismatches.

## 6 Conclusion

The relationship between financial deepening and corporate healthy development has long been a focus of theoretical research. In the wake of the financial crisis, scholars have increasingly discussed the economic consequences of financialization, with the aim of clarifying the intrinsic relationship and interaction between financial markets and the real economy. This article empirically tests the impact of investment income and fixed income financialization on the term structure of investment and financing, and further analyzes the above causal relationship according to the different characteristics of enterprises. Finally, the change of China's external financial regulatory environment is used to test the moderating effect of the main causality.

The results show that: first, there is an inverted U-shaped nonlinear relationship between the financialization of investment income and fixed income and “maturity mismatch”. The term mismatch of investment and financing increases with the degree of financialization, after reaching the critical point, it eases with the deepening of financialization. However, the specific point of view is different. In the sample interval, the investment income financialization exacerbates the investment and financing maturity mismatch more obviously; the fixed income financialization inhibits the investment and financing maturity mismatch more obviously.

Second, heterogeneity analysis shows that: the non-state enterprise's allocation of investment income financial assets is more sensitive to investment and financing maturity mismatch than the state enterprise; the state enterprise's allocation of fixed income financial assets is more sensitive to the impact of investment and financing maturity mismatch than non-state enterprises. Enterprises with high financing constraints and low managerial agency conflict are more sensitive to the impact of the allocation of two types of financial assets on investment and financing maturity mismatch than enterprises with low financing constraints and serious managerial agency conflict.

Third, the regulatory effect of financial supervision weakens the inverted U-shaped relationship between investment income financialization and investment and financing term mismatch; it enhances the inverted U-shaped relationship between fixed income financialization and investment and financing maturity mismatch. In general, financial supervision has had a significant positive effect on investment and financing maturity mismatches.

The findings of this paper verify the double-edged sword effect triggered by different motives for financialization, examine the effects of different motives on the maturity structure of funds, enrich the relevant theories on the motives for financialization and investment and financing maturity mismatches, and fill in the research gaps in the relationship between the two mentioned above; The study validates the passivity hypothesis of the existing literature on investment and financing maturity mismatch of Chinese firms, provides real-world empirical evidence for understanding the relationship between financialization and investment and financing maturity mismatch, and is able to better enable stakeholders to understand financialization, demonstrating its contribution to academia; Based on the background of Chinese characteristics, this paper utilizes the changes in financial regulatory policies to further study the above major causal relationships, which enriches China's practice in the financial field and provides a theoretical basis and a direction of thinking for future financial regulatory reform. In addition, there are still certain shortcomings in the research, such as the study of the mechanism of action, the study of the appropriateness of financialization, and the study of the economic consequences of financialization in different economic cycles, which still need to be explored in depth.

The research in this article has important policy implications.

Firstly, it is necessary to improve the willingness and confidence of enterprises to invest in entities and encourage investment in the real economy. Local governments should play a good service function, effectively implement preferential support policies for the real industry, and create a good business environment. The board of directors, supervisory boards, external investors and other relevant bodies should play a good internal governance and external supervision functions, innovate supervision and incentives, optimize investment and financing decisions, and improve the efficiency of corporate governance as a whole.

Secondly, it is necessary to smooth direct and indirect financing channels for enterprises and improve the financial inhibition environment. We should improve the construction of China's multi-level capital market system, deepen the reform of the registration system, and broaden the market's long-term capital supply channels. Efforts should be made to solve the problem of credit discrimination by formal financial institutions, rationally optimize the structure of bank credit fund supply, improve the term risk pricing mechanism of bank loans, and increase the willingness of banks to provide long-term loans.

Thirdly, financial regulation should be strengthened to reduce systemic financial risks. The construction of a unified regulatory system should be strengthened and regulatory shortcomings should be made up, while risk management and prevention should be carried out with regard to the characteristics of different types of enterprises. In addition, it is necessary to guard against the excessive and disorderly development of private finance, regulate and guide alternative financing channels in an orderly manner, and prevent shadow banking risks.

## Data availability statement

The data will be available on request from corresponding author.

## CRediT authorship contribution statement

**Hongwen Jia:** Writing – original draft, Validation, Project administration, Funding acquisition, Data curation. **Nan Gao:** Writing – review & editing, Software, Methodology, Formal analysis. **Fayyaz Ahmad:** Writing – original draft, Investigation, Conceptualization. **Ahsan Farooq:** Validation, Investigation. **Aamir Javed:** Writing – review & editing, Writing – original draft, Visualization, Investigation.

## Declaration of competing interest

The authors declare that they have no known competing financial interests or personal relationships that could have appeared to influence the work reported in this paper.
